# The effect of azithromycin for management of HIV-associated chronic lung disease on right heart function: Results from the BREATHE trial

**DOI:** 10.1016/j.ijcha.2021.100920

**Published:** 2021-11-20

**Authors:** Edith D. Majonga, Gugulethu Newton Mapurisa, Andrea M. Rehman, Grace McHugh, Tsitsi Bandason, Hilda Mujuru, Carmen Gonzalez-Martinez, Jon O. Odland, Neil Kennedy, Rashida A. Ferrand

**Affiliations:** a Biomedical Research & Training Institute, Harare, Zimbabwe; b Department of Medical Physics & Imaging Sciences, University of Zimbabwe, Harare, Zimbabwe; c Department of Paediatrics and Child Health, University of Malawi College of Medicine, Blantyre, Malawi; d MRC International Statistics and Epidemiology Group, Department of Infectious Disease Epidemiology, London School of Hygiene & Tropical Medicine, London, UK; e Department of Paediatrics, University of Zimbabwe, Harare, Zimbabwe; f Malawi-Liverpool-Wellcome Trust Clinical Research Programme, Blantyre, Malawi; g Faculty of Health Sciences, UiT, The Arctic University of Norway, Tromsø, Norway; h School of Health Systems and Public Health, Faculty of Health Sciences, University of Pretoria, Pretoria, South Africa; i Centre for Medical Education, Queen’s University, Belfast, UK; j Department of Clinical Research, London School of Hygiene & Tropical Medicine, London, UK

**Keywords:** Children, Adolescents, Africa, HIV, Chronic lung disease, Right heart

## Abstract

**Background:**

Right heart abnormalities and pulmonary hypertension (PH) may be secondary to chronic lung disease. Chronic lung disease is common in children with HIV. In the BREATHE trial (***Trial registration:*** NCT02426112), azithromycin (AZM) reduced the risk of acute respiratory exacerbations in children aged 6–19 years with HIV-associated chronic lung disease (HCLD) taking antiretroviral therapy. We assessed the possible effect of AZM on right heart dysfunction and/or PH in the trial.

**Methods:**

A standardised transthoracic echocardiogram using M-mode, two-dimensional and Doppler was performed, at baseline and at completion of weight-based AZM given weekly for 48 weeks. Linear regression was used to compare trial arms.

**Results:**

A total of 169 participants (82 AZM arm; 87 placebo arm) were included. Participants in the placebo arm were older, median age 16.2 (13.0–18.2) vs 15.3 (12.9–17.4) years, p = 0.184 in the AZM arm. At baseline, right heart abnormalities (right ventricular systolic dysfunction (RVSD), dilatation, or PH) were observed in 7(4%). Following treatment, there was no difference in prevalence of RVSD between arms (p = 0.761). There was one incident case of suspected PH, and overall, no difference in pulmonary pressures.

**Conclusion:**

In children with HCLD, there was evidence of secondary cardiac effects, but AZM had no effect on right heart function. Long-term follow-up in children with HIV should be part of future research to understand the clinical implications of right heart abnormalities.

## 1 Introduction

Recent studies have shown a high prevalence and incidence of structural and functional echocardiographic abnormalities among children with HIV [1], [2], [3]. Right heart abnormalities may be secondary to chronic lung disease (CLD) [4] which is common among children with HIV [5]. Pulmonary hypertension (PH) can develop as a complication of CLD causing right ventricular (RV) remodelling and subsequent right heart failure [4]. Impaired RV structure and/or function is associated with poor clinical outcomes [6].

Azithromycin (AZM) has both antibiotic and immunomodulatory properties and is used in management of a variety of chronic lung diseases. We conducted a trial to investigate the impact of AZM on lung function and acute respiratory exacerbations (ARE) among children with HIV-associated CLD. We hypothesized that any beneficial effects on lung function and/or risk of ARE could have a secondary effect on right heart function. In this paper, we report the findings of a sub-study to investigate the effect of AZM on prevalence of right heart dysfunction and PH.

## 2 Methods

The BREATHE trial (Bronchopulmonary function in response to azithromycin treatment for CLD in HIV-infected children, *trial registration NCT02426112*) is an individually-randomised double-blind, placebo-controlled trial conducted in Malawi and Zimbabwe. The trial protocol has been published elsewhere [7]. Briefly, children aged 6–19 years, taking antiretroviral therapy (ART) for at least 6 months with CLD (defined as forced expiratory volume in one second (FEV1) z-score of −1 and lack of reversibility with salbutamol) were recruited from outpatients HIV clinics from two public-sector hospitals in Harare, Zimbabwe and Blantyre, Malawi. Trial participants were randomised to receive weight-based weekly AZM or placebo for 48 weeks. Ethical approval was obtained from Medical Research Council of Zimbabwe, College of Medicine Research Ethics Committee and the London School of Hygiene and Tropical Medicine and the University of Tromso.

Transthoracic echocardiography was performed to assess RV size and function and PH, at baseline and at 48 weeks. Echocardiography was performed according to American Society of Echocardiography guidelines [8]. The RV basal diameter, tricuspid annular plane systolic excursion (TAPSE) and right atrial (RA) area were measured. Tricuspid peak gradient and pulmonary arterial systolic pressure (PASP) were also estimated [9]. RV and RA dilatation were defined as > +2 z-score of RV diameter and RA area respectively, using European references [10]. RV systolic dysfunction (RVSD) was defined as < -2 z-score of TAPSE using reference equations from Zimbabwean children [11]. PH was defined as tricuspid regurgitation velocity ≥ 2.9 m/s and PASP ≥ 37 mmHg (assuming an RA pressure of 5 mmHg) [9].

Due to technical issues with the ultrasound machine in Malawi, the current sub-study could not be conducted in Malawi. Therefore, this study was conducted in Zimbabwe only.

### 2.1 Statistical analysis

Analyses were performed using Stata v15·0 software (StataCorp, Texas, USA). Continuous data was presented as mean ± standard deviation (SD) if they were normally distributed or median (interquartile range, IQR) if not normally distributed. For categorical variables, the number and percentage in each category was reported. All continuous data were tested for heteroskedasticity and corrected for, prior to any regression analysis. Heteroskedasticity was assessed through visual inspection of residuals vs fitted values plots and Cook-Weisberg test. The presence of heteroskedasticity was addressed by use of robust standard errors.

Linear regression was used to compare cardiac measures in the trial arms, to estimate the mean difference and corresponding 95% confidence interval (CI), adjusting for baseline value of the cardiac measure as a continuous covariate, categorical age, sex, and log 10 viral load. The mean change in z-scores in each trial arm for the primary cardiac measures was calculated as mean z-score (12 months) minus mean z-score (baseline) and adjusted for baseline z-scores, age, sex and viral load using linear regression. Multivariable logistic regression was used to assess which covariates were associated with right heart abnormalities at baseline.

A total of 18 (10%) scans at baseline were used to estimate intra-rater variability for RV and RA measures using intra-class correlation coefficient (ICC). ICC values range from 0 to 1 with values above 0.75 representing good to excellent reliability, and values below 0.75 representing moderate to poor reliability.

## 3 Results

**Fig. 1:**
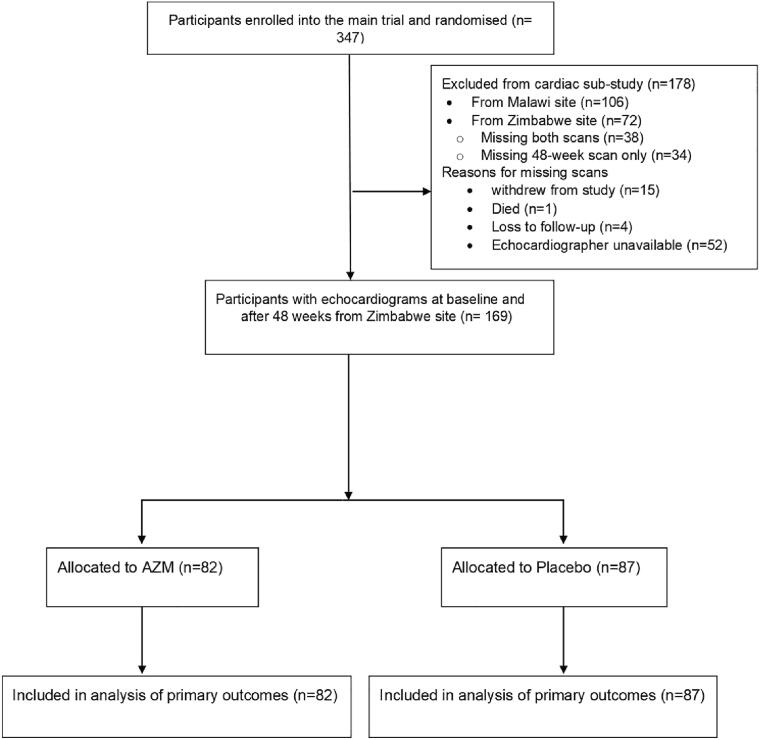
Study flow diagram.

Of the 347 participants enrolled in the trial, 241 were enrolled from Zimbabwe and of these 169 (82 AZM arm; 87 placebo arm) were included in this sub-study. Participants excluded were missing either a baseline or a 48-week echocardiogram (Fig. 1). In those excluded (n = 72), there were no age or sex differences to those included the study. Participants in the placebo arm were older, median age 16.2 (13.0–18.2) vs 15.3 (12.9–17.4) years, , p = 0.184 in the AZM arm (Table 1).

**Table 1:** Baseline clinical characteristics of participants.

Characteristic	Azithromycin arm N = 82	Placebo arm N = 87	P-value
**Demographic**			
Sex, female, N (%)	33 (40)	40 (46)	0.452
Age, y, median (IQR)	15.3 (12.9–17.4)	16.2 (13.0–18.2)	0.184
**HIV**			
HIV VL, median log10 copies/ml, median (IQR)	2.6 (1.8–3.8)	2.9 (2.1–4.2)	0.151
VL < 1000 copies/ml, N (%)	50 (61)	48 (55)	0.445
CD4 count, (cells/mm^3^), median (IQR)	571 (384–736)	508 (278–740)	0.354
CD4 count < 100, (cells/mm^3^), N (%)	6 (7)	6 (7)	0.915
Age at HIV diagnosis, y, median (IQR)	7 (3–9)	8 (5–11)	0.061
Age at ART start, y, median (IQR)	7 (4–10)	9 (6–12)	**0.034**
Duration on ART, y, median (IQR)	4 (3–4)	4 (2–4)	0.307
**ART Regimen, n (%)**			0.057
EFV/NVP	49 (60)	64 (74)	
ATV/LPV	33 (40)	23 (26)	
Taking cotrimoxazole prophylaxis, n (%)	74 (90)	79 (91)	0.901
**Clinical**			
Body surface area, m^2^, mean (SD)	1.24 (0.23)	1.30 (0.24)	0.081
Weight-for-age z-score, mean (SD)	−2.29 (1.43)	−2.04 (1.57)	0.294
Underweight, n (%)	45 (55)	41 (47)	0.314
Height-for-age z-score, mean (SD)	−2.10 (1.14)	−1.92 (1.26)	0.346
Stunted, n (%)	42 (51)	35 (40)	0.152
Respiratory rate, breaths/minute, mean (SD)	22 (3)	22 (3)	0.668
Heart rate, beats/minute, mean (SD)	86 (13)	85 (11)	0.448
Oxygen Saturation, (%), mean (SD)	96 (3)	97 (3)	0.659

VL, viral load; ART, antiretroviral therapy; AZM, azithromycin; SD, standard deviation; IQR, inter-quartile range; EFV, efavirenz; NVP, nevirapine, ATV, atazanavir; LPV, lopinavir.

At baseline, right heart abnormalities (RVSD, RV dilatation, or PH) were observed in 7(4%) participants. Mean RV, RA and TAPSE z-scores were similar between the arms at baseline and 48 weeks (Table 2). After treatment with AZM, 4(5%) vs 3 (4%) of participants in the placebo and AZM arm respectively had RVSD (p = 0.761), of which one participant had RVSD at both baseline and 48 weeks. At 48 weeks, PH was suspected in in two participants, one in the placebo and AZM arm, respectively. Mean RV z-scores following treatment were higher in the placebo arm, +0.43 (0.8) compared to + 0.36 (0.6) in the AZM arm, but not significantly so - adjusted mean difference of −0.05 (95% CI using robust standard errors, −0.20 to 0.09, p = 0.488). No clinical or HIV related factors were associated with right heart abnormalities. Left ventricular systolic function was similar between trial arms.

**Table 2:** Echocardiographic Findings at baseline and after 48 weeks.

	Baseline		48 weeks		Adjusted mean difference between arms (95% CI) a	P-value
Cardiac parameters	AZM (N = 82)	Placebo (N = 87)	AZM (N = 82)	Placebo (N = 87)		
**Right**						
RV diameter, mm b, mean (SD)	35.6 (3.6)	36.4 (3.9)	37.1 (3.7)	38.2 (3.9)	−0.21 (−0.78–0.36)	0.469
RV diameter z-score, mean (SD)	0.22 (0.7)	0.21 (0.6)	0.36 (0.6)	0.43 (0.8)	−0.05 (−0.20–0.09)	0.488
RA area cm^2^^b^, mean (SD)	11.6 (2.1)	11.8 (2.3)	12.6 (2.3)	12.8 (2.4)	−0.22 (−0.64–0.21)	0.312
RA area z-score, mean (SD)	0.16 (0.9)	−0.06 (0.8)	0.34 (0.8)	0.23 (0.8)	−0.05 (−0.23–0.14)	0.625
TAPSE mm b, mean (SD)	20.6 (2.2)	20.8 (2.2)	20.3 (2.2)	20.6 (0.9)	−0.28 (−0.98–0.38)	0.398
TAPSE z-score, mean (SD)	−0.15 (1.0)	−0.11 (0.9)	−0.32 (1.0)	−0.26 (0.9)	−0.11 (−0.39–0.18)	0.454
Tricuspid peak gradient, mmHg b, median (IQR)	5.3 (3.9–10)	4.7 (3.5–10)	5.4 (3.7–11.5)	6.4 (3.9–12.0)		
PASP mmHg, median (IQR)	10.4 (8.9–15)	10.0 (8.5–15.7)	10.7 (8.7–18.9)	12.0 (8.9 –17.8)		
PH, N (%)	1(1)	–	1(1)	1(1)		
RV dilatation, N (%)	1(1)	–	–	–		
RVSD, N (%) c	3 (4)	2(2)	3 (4)	4 (5)		
**Left**						
LV diameter z-score	0.46 (1.5)	0.69 (1.4)	0.80 (1.4)	0.91 (1.4)	0.13 (−0.11–0.38)	0.285
IVS diameter z-score	0.58 (1.4)	0.36 (1.5)	0.92 (1.4)	1.00 (1.5)	−0.28 (−0.60–0.05)	0.094
LVPW diameter z-score	0.12 (1.2)	−1.73 (1.3)	0.23 (0.9)	0.17 (1.3)	−0.04 (−0.35–0.27)	0.805
LV ejection fraction, %	62.6 (6.2)	63.0 (6.7)	61.6 (6.2)	61.9 (6.2)		
**Cardiac symptoms**						
Tachycardia, N (%)	2 (2)	1(1)	1 (1)	3 (3)		NS
Tachypnoea, N (%) d	8 (10)	7 (8)	12 (15)	9 (10)		NS
Hypoxia, N (%)	2 (2)	5 (6)	2 (2)	–		NS
Shortness of breath, N (%)	2 (2)	1 (1)	1(1)			NS

AZM, azithromycin; RV, right ventricular; RA, right atrium; RVSD, right ventricular systolic dysfunction; TAPSE, tricuspid annular plane systolic excursion; PASP, pulmonary arterial systolic pressure; LV, left ventricular; IVS, interventricular septum; LVPW, left ventricular posterior wall; SD, standard deviation; IQR, inter-quartile range; NS, not significant

a Mean difference comparing arms at 48 weeks adjusted for baseline cardiac measure, age category, sex, and viral load log10 copies/ml.

b Missing values: RV diameter n = 3; TAPSE n = 2; RA diameter n = 10; Tricuspid peak gradient n = 9.

c n = 1 had RVSD at baseline and 48 weeks.

d n = 3 had tachypnoea at baseline and 48 weeks.

The ICC analysis for RV basal diameter was (0.921), TAPSE (0.878) and for RA area (0.879), Overall ICC showed good intra-rater reliability.

## 4 Discussion

The study found that 4% of participants had right heart abnormalities and were not associated with elevated right heart pressures. PH was rare at both baseline and 48 weeks. Although echocardiography is a recommended screening tool for suspected PH, doppler echocardiography can frequently under-or overestimate pulmonary arterial pressures in patients [12]. Importantly, other echocardiographic signs of pH which may support suspicion of pH appeared to be absent or quite minimal in this study including enlarged right atrium, systolic flattening of the interventricular septum and RV dilatation. This may further support the rarity of this condition in this study. RV remodelling is typically secondary to increase in RV afterload but may occur in the absence of elevated pulmonary pressures [13].

CLD may induce altered structure and function of the right heart due to abnormal pulmonary function and this is often associated with PH. While AZM was associated with a reduction of risk of acute respiratory exacerbations [14], it had no effect on pulmonary function and right heart abnormalities. It is possible that the mechanism of the observed right heart abnormalities was not due to lung function impairment but, HIV *per se* may have induced the changes. We have previously reported an 8% prevalence of RV abnormalities in a cohort of children established on ART, more than half of whom had concurrent left heart abnormalities, suggesting a cardiomyopathy affecting both sides of the heart [2]. It is also plausible that ART and viral suppression play a significant role in preventing clinical cardiomyopathy. All participants were on ART but only 58% were virally suppressed at enrollment. We acknowledge that follow-up time may have been too short to observe any beneficial effects of AZM on cardiac disease, and/or insufficient power to detect any potential effects.

Limitations of this study include lack of right heart catheterisation to completely rule- out PH. There is possible underestimation of pulmonary pressures due to the inherent limitations and operator dependency of the echocardiography technique. Importantly, this study was performed in a resource-limited setting and more advanced contemporary imaging measures were not available including three-dimensional echocardiography, tissue doppler and speckle tracking imaging. Their use may have provided better imaging of global or regional function. However, we only used optimum images to acquire measurements from in this study. Notably, in most healthcare settings in this region, there is not widespread availability of advanced echocardiographic techniques and so our findings will be more readily applicable to a low-resource setting, which is where the majority of these patients are seen.

In summary, right heart abnormalities were observed in a small proportion of children with HCLD taking ART, but AZM had no impact on right heart size and function. Long-term follow-up in children with HIV should be part of future research to understand the clinical implications of right heart abnormalities.

## Funding

The study was funded through the Global Health and Vaccination Programme of the Norwegian Research Council.

## Declaration of Competing Interest

The authors declare that they have no known competing financial interests or personal relationships that could have appeared to influence the work reported in this paper.
